# Identification of miR-379/miR-656 (C14MC) cluster downregulation and associated epigenetic and transcription regulatory mechanism in oligodendrogliomas

**DOI:** 10.1007/s11060-018-2840-6

**Published:** 2018-06-21

**Authors:** Anupam Kumar, Subhashree Nayak, Pankaj Pathak, Suvendu Purkait, Prit Benny Malgulawar, Mehar Chand Sharma, Vaishali Suri, Arijit Mukhopadhyay, Ashish Suri, Chitra Sarkar

**Affiliations:** 1 Department of Neurosurgery, Neurosciences Centre, All India Institute of Medical Sciences - AIIMS, New Delhi, India; 2 Department of Pathology, All India Institute of Medical Sciences, Ansari Nagar, New Delhi, 110029 India; 3 Genomics and Molecular Medicine, CSIR-Institute of Genomics and Integrative Biology, Room No. 331, Mathura Road (near Sukhdev Vihar), New Delhi, 110020 India; 4 School of Environment and Life Sciences, University of Salford, Room 203a, Cockcroft Building, Manchester, M5 4WT UK

**Keywords:** miRNA cluster, C14MC, MEG3, MEF2, Oligodendrogliomas

## Abstract

**Introduction:**

Although role of individual microRNAs (miRNAs) in the pathogenesis of gliomas has been well studied, their role as a clustered remains unexplored in gliomas.

**Methods:**

In this study, we performed the expression analysis of miR-379/miR-656 miRNA-cluster (C14MC) in oligodendrogliomas (ODGs) and also investigated the mechanism underlying modulation of this cluster.

**Results:**

We identified significant downregulation of majority of the miRNAs from this cluster in ODGs. Further data from The Cancer Genome Atlas (TCGA) also confirmed the global downregulation of C14MC. Furthermore, we observed that its regulation is maintained by transcription factor MEF2. In addition, epigenetic machinery involving DNA and histone-methylation are also involved in its regulation, which is acting independently or in synergy. The post- transcriptionally regulatory network of this cluster showed enrichment of key cancer-related biological processes such as cell adhesion and migration. Also, there was enrichment of several cancer related pathways viz PIK3 signaling pathway and glioma pathways. Survival analysis demonstrated association of C14MC (miR-487b and miR-409-3p) with poor progression free survival in ODGs.

**Conclusion:**

Our work demonstrates tumor-suppressive role of C14MC and its role in pathogenesis of ODGs and therefore could be relevant for the development of new therapeutic strategies.

**Electronic supplementary material:**

The online version of this article (10.1007/s11060-018-2840-6) contains supplementary material, which is available to authorized users.

## Introduction

Oligodendroglioma (ODG) accounts for 5–9% of all gliomas [1] (CBTRUS statistical report: NPCR and SEER; 2006–2010) and is classified by World Health Organization (WHO, 2016) into IDH1 mutant and 1p/19q co-deleted grade II and IDH1 mutant and 1p/19q co-deleted anaplastic grade III ODGs [2]. Emerging evidences suggest significant role of dysregulated microRNAs (miRNAs) in the pathogenesis of cancers. MiRNAs have been established as critical players in tumor progression and potent candidates in the advancement of cancer diagnostics and therapeutics [3, 4]. These short non-coding RNA molecules usually negatively regulate the translation of target mRNA by binding to their 3′untranslated region (3′UTR) [5]. Due to its short length and imperfect base-pairing, an individual miRNA is predicted to have hundreds of target mRNAs. Conversely, several miRNAs are grouped in cluster and regulate a single target. Studies accumulated in recent pasts have identified the complex role of miRNA clusters transcribed in the human genome and several reports have linked clustered miRNAs to variety of cancers, in which these shown to act like either oncogenes or tumor suppressors [6]. Though there are few reports demonstrating the role of individual miRNAs in pathogenesis of ODGs are well documented [7, 8] however, the contribution of clustered miRNAs remains unexplored.

miR-379/miR-656 cluster [hereafter named as C14MC], one of the largest miRNA clusters, is located within an imprinted chromosomal region DLK1(delta-like homolog 1)—DIO3(iodothyronine deiodinase 3) on chromosome 14q32.31 region and harbors distinct imprinted genes (*DLK1, RTL1, MEG3, MEG8* and *DIO3*), C/D small nucleolar RNAs (*SNORDs*) and more than 50 miRNA genes [9, 10]. The polycistronic nature of this cluster has been shown to be under positive regulation of Mef2 transcription factor in rat neurons [11].

Several studies have documented dysregulation of this cluster in various cancers such as melanoma, ependymoma, medulloblastoma, neuroblastoma and lung adenocarcinoma [12–16]. However, its role in the oligodendrogliomas remains poorly understood. Therefore, in the present study, we analyzed the expression of several miRNAs from C14MC cluster and identified their downregulation in ODGs. In addition, we also investigated the possible functional mechanism for its downregulation. Our study showed that transcription factor MEF2, along with epigenetic machinery involving hypermethylation of the cluster locus and enrichment of H3K27me3 mark at the regulatory region could be the underlying mechanism for the downregulation of this cluster which either acts independently or in synergy. Further downregulation of this cluster also showed prognostic significance in ODGs.

## Materials and methods

### Patient characteristics

The study included 43 cases of oligodendroglioma showing classical histology and also fulling recent WHO 2016 classification by showing co-deletion of 1p/19q and IDH/2 mutations and retained ATRX expression. There were 23 cases of Grade II (MIB1-1 to 12%) and 20 cases of grade III (MIB1-5 to 28%) with mean age of 40 years ranging from 26 to 65 years with M:F ratio of 3.4:1. A summary of the clinical features is shown in Supplementary Table S1.

### Nucleic acid isolation

Total DNA and RNA from frozen tumor and control tissues were extracted using Qiagen mini DNA prep (Qiagen, Germany) and mirVana total RNA isolation Kit (Ambion, USA), respectively, following the manufacturer’s instructions. Concentration and purity of the total DNA and RNA samples were measured using the Picodrop Microliter UV/Vis Spectrophotometer (Picodrop Limited, UK) and gel electrophoresis.

### Analysis of co-deletion of 1p/19q, IDH1 mutation

Co-deletion of 1p/19q in all the ODGs was assessed by dual-probe fluorescence in situ hybridization (FISH) assay as per our standard protocol described before [17]. Mutations in exon 4 of *IDH1* gene was determined using direct bidirectional sequencing as described before [17], Supplementary Table S2.

### C14MC expression profiling by TLDA

miRNA profiling for the C14MC microRNA cluster was performed using customized TaqMan low density array (TLDA) microfluidic cards (Applied Biosystems, CA) as described earlier [23]. Details of the candidate miRNAs and endogenous controls found on TLDA cards are provided in Supplementary Table S3. Threshold cycle (Ct) values greater than 35 were imputed to 35 according to the technical recommendation [24]. The Ct was calculated by relative quantification using 2^−∆∆Ct^ method [18].

### C14MC expression analysis using TCGA data

Genome-wide small RNA sequencing level 3 data (calculated expression for all reads aligning to a particular miR, per sample) for 153 ODGs samples (Grade II-64, Grade III-89) and 5 controls was extracted from The Cancer Genome Atlas (TCGA) and was analyzed for differentially expressed miRNAs from C14MC cluster. Differential expression analysis was performed using non-parametric two-tailed Mann–Whitney *U* test and corrected for multiple comparisons by Bonferroni correction [19].

### cDNA conversion and RT-PCR based gene expression analysis

Expression of all the genes and miRNAs of interest were studied by real-time RT-PCR. 10 ηg of cDNA was used for qPCR reaction for all the genes of interest SnU6 and GAPDH were used as endogenous control for miRNA and mRNA, respectively. The primer sequences of the genes and miRNAs are listed in Supplementary Table S2. The Ct was calculated by relative quantification using 2^−∆∆Ct^ method [18].

### Cell culture and 5-azacytidine treatment

Oligodendroglioma cell line HS683 [20] was obtained from ATCC (HTB-138™) and cultured in Dulbecco’s modified Eagle medium (DMEM) supplemented with 10% fetal calf serum, 0.2 mM glutamine and antibiotics (50 µg/ml penicillin, 50 µg/ml streptomycin) and incubated at 37 °C in an atmosphere containing 5% CO_2_. For drug treatment cells were seeded at a density of 5 × 10^5^ cells per well in six-well plate and treated 5-azacytidine (Sigma, USA) at a concentration of 5 µM and the medium was replenished every 24 h until the 72-h treatment was completed. Expression of miRNAs and MEG3 was analyzed using qRT-PCR.

### Chromatin immunoprecipitation (ChIP)-qPCR

ChIP assays were performed using the Low Cell ChIP Kit (M/S Diagenode, Belgium) protocol with some minor modifications. Briefly, cell pellets were collected and cross-linked using 1% formaldehyde in PBS and reaction was stopped using 100 µl of 125 mM glycine. The chromatin was fragmented using Bioruptor plus (M/S Diagenode, Belgium) and fragmented chromatin (equivalent to 1 million cells) was immunoprecipitated using anti-MEF2A antibody (M/s Abcam, USA), anti-H3K27me3 antibody (M/s Abcam, USA), anti-IgG Mouse (M/s Abcam, USA) and Rabbit antibody (M/s Abcam, USA) as per Low Cell ChIP kit guidelines. After overnight incubation, the agarose beads were washed and immunoprecipitated and input DNA was processed for DNA isolation using IPure kit (M/s Diagenode, Belgium) following the manufacturer’s instructions. The precipitated DNA fractions were quantified by real-time RT-PCR using SYBR Green dye with primers encompassing binding site region of MEF2A, MEG3 and GAPDH (negative control). The primer sequences are listed in Supplementary Table S2. Target **e**nrichment was expressed as the percent input by using the following formula: Percentage of total input = 100 × 2^[Ct (ChIP)–(Ct input–log2 (input dilution factor))] [21].

### Methylation status analysis of C14MC

Genome wide methylation data generated on Infinium HumanMethylation450K BeadChip (Illumina Inc.) was obtained for 156 ODG cases from TCGA data portal and analysis was done as described previously [19]. Methylation status of MEG3 promoter was tested using methylation-specific PCR primers designed for MEG3-DMR in ODGs. The primer sequences are listed in Supplementary Table S4.

### Genome-wide mRNA expression analysis

The mRNA expression profiles of 13 ODGs (6 Grade II, 7 Grade III) and 3 normal brains were examined using Affymetrix Human 2.0 ST array (Affymetrix Inc, CA) as per the protocols outlined in detail in the geneChip@ expression analysis technical manual (Affymetrix, http://www.affymetrix.com/support/downloads/manuals/expression_analysis_technical_manual.pdf). mRNA signal intensities were log2 transformed and analyzed for differentially expressed mRNAs using the Transcriptome Analysis Console (TAC) (Affymetrix Inc, CA) [22]. Differentially detected mRNA signals with ≥ 2.0 fold-change and the P < 0.05 were considered significant.

### Survival analysis

The prognostic significance of miRNAs from C14MC cluster was analyzed using TCGA clinical dataset. Of the 156 TCGA ODG samples, survival data was available for 82 ODGs. The data was partitioned into two quantiles and Kaplan–Meier survival analysis was used to examine progression-free survival (PFS) and comparison was performed by log-rank test.

### Statistical analysis and bioinformatic analysis

Statistical analysis was conducted with the SPSS software version 16.0 (SPSS, Inc., Chicago, IL, USA) for clinicopathological correlations. TCGA and REMBRANDNT dataset were used as an independent data set for experimental results validation on larger sample set. P-values < 0.05 obtained using *t*-test were considered statistically significant.

## Results and discussion

### C14MC cluster are downregulated in oligodendrogliomas

**Fig. 1:**
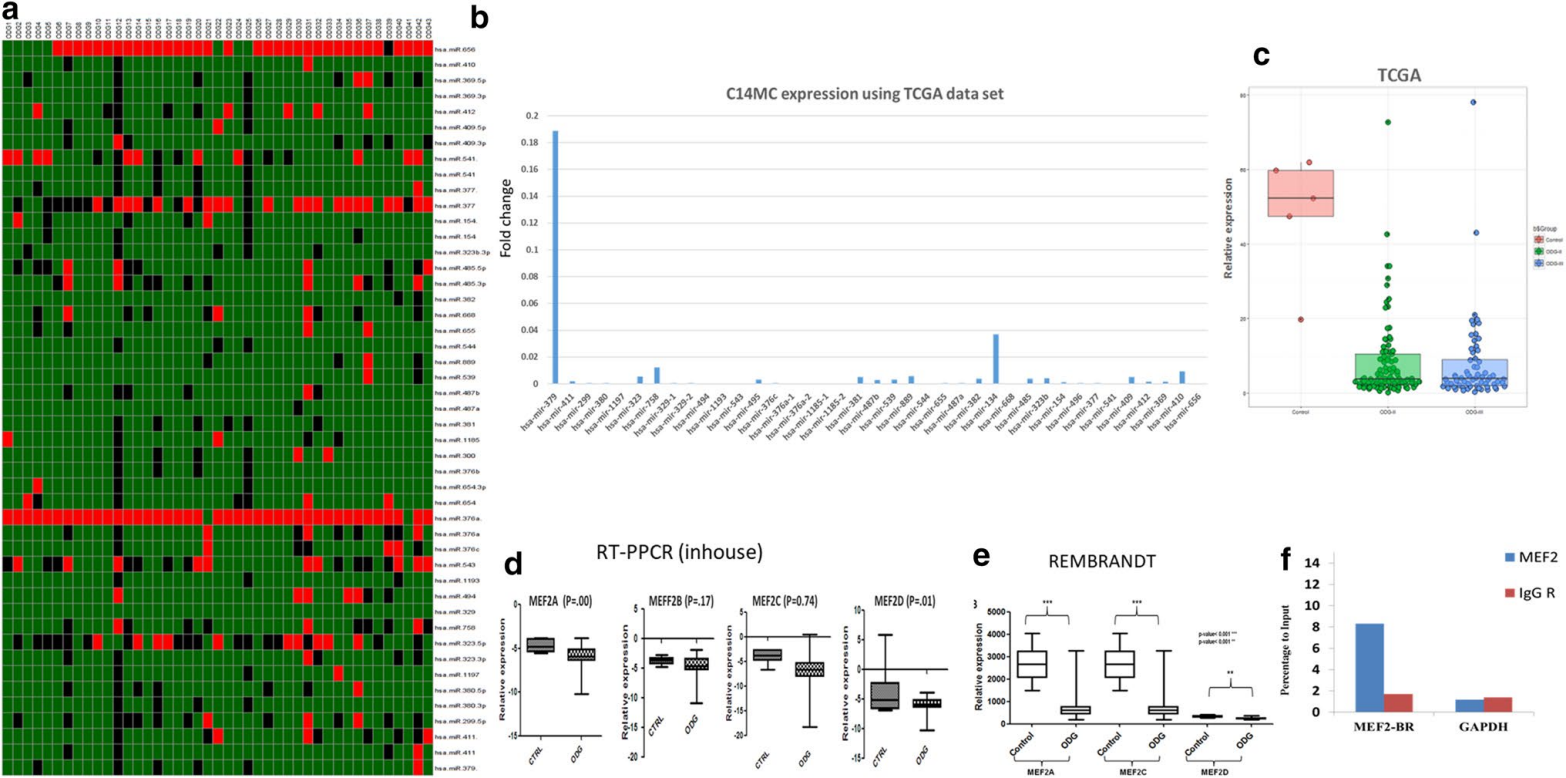
**a** The heat map shows the fold changes of miRNAs of C14MC in 43 ODGs with respect to normal brain using TLDA. Fold change ≤ 0.5 represents down-regulation (green), ≥ 2 upregulation (red) and > 0.5 to < 2 non-differential expression (Black). **b** Bar graph showing homogenous down-regulation of C14MC in ODG samples as compared to controls. **c** Box plot showing differential expression C14MC between grade-II vs grade-III ODGs using TCGA dataset. **d** Box plot shows expression levels of different *MEF2* isoforms. “CTRL” and “ODG” represent controls and patient data, respectively. Significant downregulation was found for *MEF2B* and *MEF2D*. **e** The normalized expression values of each isoforms of MEF2 from REMBRANDANT dataset. Y-axis shows expression levels of different *MEF2* isoforms in ODGs. **f** ChIP-qPCR showing binding of MEF2A to the MEF2 binding site (MEF2-BR) upstream of C14MC cluster using anti-Mef2A antibody and IgG as a control. MEF2A ChIP assay showed eightfold enrichment of MEF2A at the MEF2 binding region

Small RNA sequencing-based expression profile for miRNAs from C14MC cluster (n = 39) in 153 ODG samples (64 Grade III and 89 Grade II) revealed global downregulation of the miRNAs (37/39 miRNAs: ~ 94%) from this cluster. Further, RT-PCR based expression analysis of 47 miRNAs from C14MC cluster using TLDA assay also showed significant downregulation of 40/47 miRNAs (Fold change < 0.6; p < 0.05) in ODG samples as compared to controls Fig. 1a, b. Majority (66%; 31/47) of the miRNAs were downregulated in more than 80% of the cases and Interestingly miR-329 was downregulated in all the cases analyzed.

On matching the TLDA and TCGA dataset, comparable downregulation was seen in majority of the miRNAs (33/39; 84%), while discordance was seen only with 6/39 (15%) miRNAs. Thus, downregulation of this cluster was established on two independent platforms and experimental setups in ODGs. Further, on comparing the expression of C14MC miRNA across the histological grades, we observed that 24% (11/47) of miRNAs (hsa-miR-154*, hsa-miR-544, hsa-miR-541, hsa-miR-409-5p, hsa-miR-329, hsa-miR-376b, hsa-miR-380-3p, hsa-miR-381, hsa-miR-654-3p, hsa-miR-369-3p, hsa-miR-487a) each p < 0.01 were differentially expressed between the grade II and III (Fig. 1c). Notably, there was higher fold under expression of miRNAs in grade III as compared to grade II. Similar to our finding, Ludwing et al. also observed more fold downregulation of several miRNAs from C14MC in grade III meningioma as compared to grade II [23]. Thus, our findings indicate that C14MC might be contributing in tumor initiation in early phase in ODGs.

### Regulation of C14MC expression

Since an orchestrated expression pattern for majority of miRNAs from C14MC throughout the samples was observed in ODGs, therefore, this further suggests either the existence of a polycystronic primary miRNA transcript or regulation of the C14MC by a common mechanism. Therefore, we investigated the underlying mechanism of C14MC downregulation. As genome level loss have been previously reported to regulate the expression of miRNAs [24] Therefore, we hypothesized that aberration of genomic region confined to C14MC could lead to their downregulation. Since, the miRNAs from C14MC are expressed only from the maternally inherited allele; deletion of the active allele may result in complete silencing of this cluster. Therefore, we performed CytoScan 750K Array (Affymetrics, USA) in representative ODG patient samples and correlated the expression of C14MC with the presence of LOH of 14q32.31. However, we did not find relevant changes in DNA copy number corresponding to the C14MC expression indicating that genomic loss is probably not responsible for reduced expression of this cluster in ODGs. This was further supported by the observation made by Lavon et al. where expression of miRNAs from the C14MC cluster was uniformly downregulated irrespective of the LOH status of 14q in GBM [25]. Hence, we propose that other mechanisms are likely responsible for silencing this cluster in ODGs.

### Transcription factor MEF2 regulates C14MC expression

MEF2 has been implicated as regulatory transcription factor of C14MC cluster in rat neuron and muscle cells [11]. Therefore, we studied the expression of all its four isoforms: *MEF2A, MEF2B, MEF2C* and *MEF2D* by real time PCR and observed significant downregulation of *MEF2A* and *MEF2D* in ODGs (p < 0.00 for both). In addition, analysis of *MEF2* expression using REMBRANDT dataset [26] also showed similar pattern of expression for MEF2 isoforms (Fig. 1d, e). Further, Since MEF2 expression correlated with C14MC expression therefore, we performed the ChIP–qPCR to confirm the binding of MEF2A on the MEF2 binding site (MBS) located upstream of the C14MC in HS683 cells. Our result showed approximately eightfold enrichment of bound chromatin compared with input confirming binding of MEF2A to the MEF2 binding site (MBS) suggesting MEF2 as a positive regulator and as a controller of C14MC expression in ODGs (Fig. 1f).

### C14MC genomic region showed hypermethylation in ODG

**Fig. 2:**
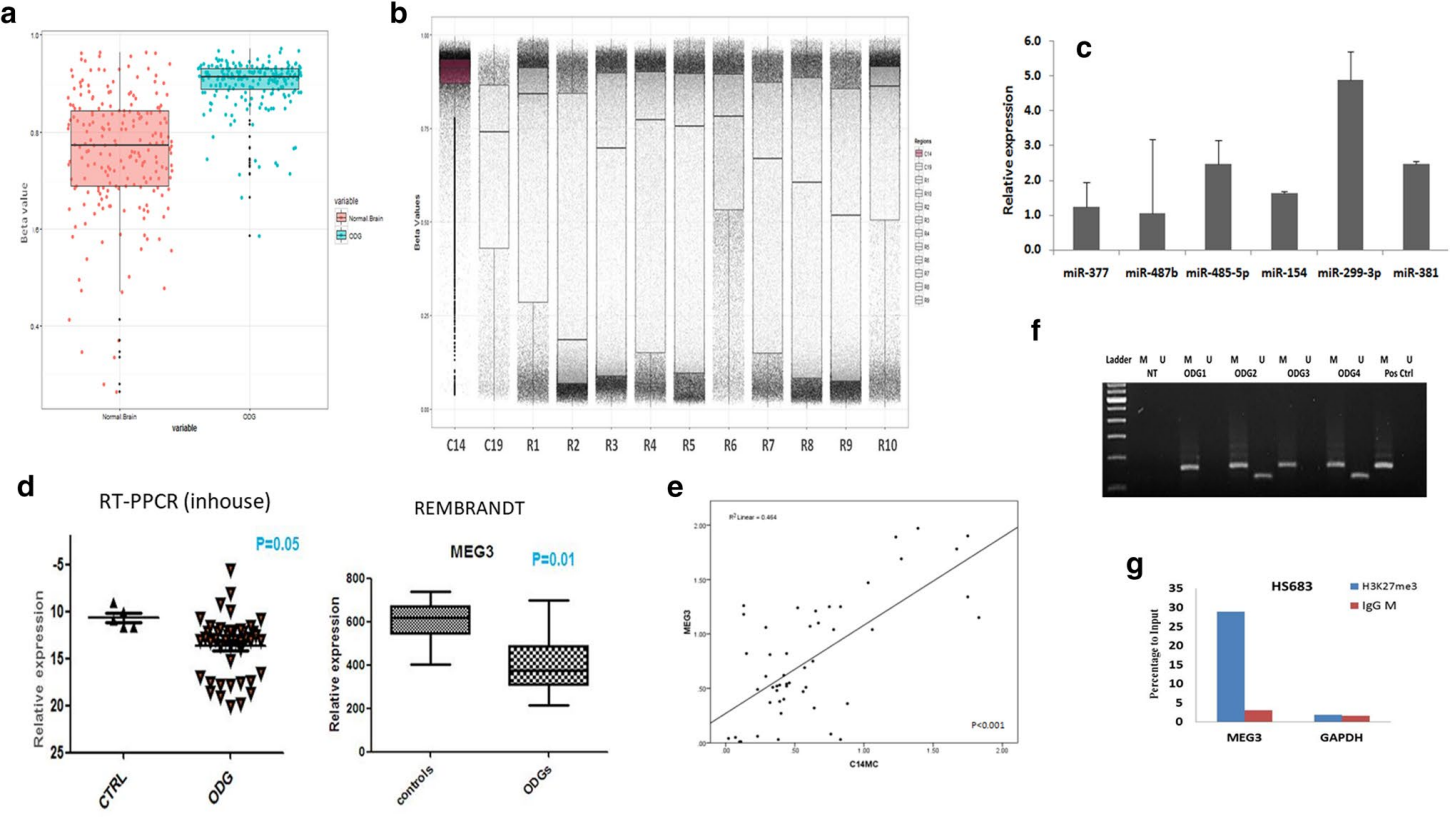
**a** Hypermethylation of C14MC domain in ODG samples as compared to normal brain as controls. Infinium HumanMethylation 450K BeadChip data (raw image files) from GEO (GSE43414) analysed using Minfi (Bioconductor package of R). Higher beta-values represent hypermethylation. **b** Relative methylation levels of the C14MC locus (~ 200 probes) are plotted along the vertical axis (beta values). The box plots determine the median beta-values of those regions. Ten random regions of the human genome (R1–R10) having similar number of methylation probes as that of C14MC region is also plotted. C19MC (miRNA cluster present on Chr19) is plotted as a control to test the specificity of the methylation pattern on C14MC in ODG. **c** Treatment with the epigenetic drugs Azacytidine (AZA) in HS683 cells showing re-expression of 3 miRNAs out of 6 C14MC miRNAs tested. Represented data is relative to control cells which were set to 1. Average of three independent experiments with standard deviation (error bars) is shown. **d** Real-time PCR data showing significant downregulation of *MEG3* (p = 0.01) in ODGs versus ctrl. REMBRANDANT dataset shows the median expressions of *MEG3* is distinctly lower in ODG than the control samples. **e** Scatter plot showing the significant correlation among the mean of miRNAs expression from C14MC cluster with MEG3 expression (R = 0.46 and P = 0.001). **f** Methylation-specific PCR assay using primer set for the methylated (M) and unmethylated (U) copies of the MEG3 DMR gene. Neg ctrl stands for the negative control (no DNA sample). Pos ctrl stands for methylated control which is universally methylated DNA. positive ctrl, ODG sample 1 and 3 were positive only for M. Samples ODG 2, 4 positive for partially methylated (M and U). A 160 bp PCR product represents the methylated state and a 120 bp PCR product stands for the unmethylated (*L* = 100 bp ladder, Invitrogen). **g** Chromatin immunoprecipitation (ChIP)-qPCR confirming enrichment of histone modifications at the promoter of the MEG3 DMR. Results of quantitative reverse transcription polymerase chain reaction (RT-PCR) conducted using DNA after ChIP with antibody against H3K27me3 showed 30 fold enrichment of bound chromatin compared with input in HS683 cell line. GAPDH was used as a control

Several studies report hypermethylation mediated silencing of most of the miRNAs from this cluster in various tumors [24, 27, 28] therefore, we analysed the methylation status of genomic locus harbouring this cluster. On comparison of methylation status of C14 locus between tumor and normal brain samples (analyzed from GEO), most of the probes for the C14MC genomic region had a median beta value of > 0.8, thus indicating this region to be significantly hypermethylated (p < 0.002) Supplementary Table S4. Further, to exclude the possibility of hypermethylation of C14MC as a random event we analysed the methylation status of ten random genomic region and observed variable distribution of beta-values (normal range; 0.2–0.8) for those regions Fig. 2a, b which confirmed it not be a random event.

Furthermore, to substantiate the role of methylation for downregulation we treated HS683 cells with DNA-demethylating agent 5-azacytidine(5′-AZA) at 5 µM for 72 h and observed restored expression for few of the randomly tested miRNAs (miR-485-5p, miR-299-3p and miR-381: p < 0.05 for each). However, some of the miRNAs tested (Mir-154, miR-487b, and miR-377) did not show change in its expression after treatment. Figure 2c These in vitro results suggest that along with DNA methylation, there exist other mechanisms of transcriptional regulation and/or posttranscriptional modification that co-operate to control C14MC expression in ODGs.

### Decreased expression of imprinted MEG3 gene mediates downregulation of C14MC

Our various aforementioned findings prompted us to consider other epigenetic explanations for the observed silencing of C14MC in ODGs. As mentioned earlier, since MEG3 and C14MC loci are in the same region therefore we studied the expression of MEG3 and correlated its expression with C14MC. Expression analysis of MEG3 using qRT-PCR and using REMBRANDT dataset showed its significant lower expression in ODG samples as compared to control (p < 0.05). Subsequently, we also observed significant correlation between MEG3 and C14MC expression (R^2^ = 0.46 and p = 0.001 using Pearson’s correlation coefficient) Fig. 2d, e.

Further, since MEG3-DMR is imprinting control center for the DLK1–DIO3 domain and it overlaps with the MEG3 promoter therefore, methylation status of MEG3 promoter was tested using methylation-specific PCR. We observed aberrant methylation of MEG3 in 37% (11/30) of cases of which, methylated pattern was seen in 13%, partially methylated in 23% whereas 63% showed unmethylated pattern Fig. 2f. Further, to investigate the role of the promoter methylation in silencing the *MEG3* gene, HS683 cells were treated with 5′-AZA at 5 µM for 72 h and showed a minimal increase in the expression of MEG3 in treated group with respect to the control group suggesting involvement of some other mechanisms other than DNA methylation for the silencing of MEG3. Therefore, we considered histone modifications for the observed downregulation of MEG3. Since H3K27me3 is associated with repressive expression, we reasoned that presence of H3K27me3 at MEG3 promoter could be associated with its silencing effect. To confirm this, Chromatin Immunoprecipitation-qPCR was performed using H3K27me3 antibody on HS683 cells and ChIP-qPCR data analysis revealed enrichment of H3K27me3 (30 fold) at MEG3-DMR suggesting histone trimethylation mediated silencing of this gene **(**Fig. 2g). Thus, our results deliver a novel mechanistic relationship of H3K27me3 in mediating gene silencing effect for MEG3 DMR in ODGs as also observed in lung adenocarcinoma and urothelial carcinoma [29, 30].

### Post-transcriptional regulatory network of C14MC in ODGs

**Fig. 3:**
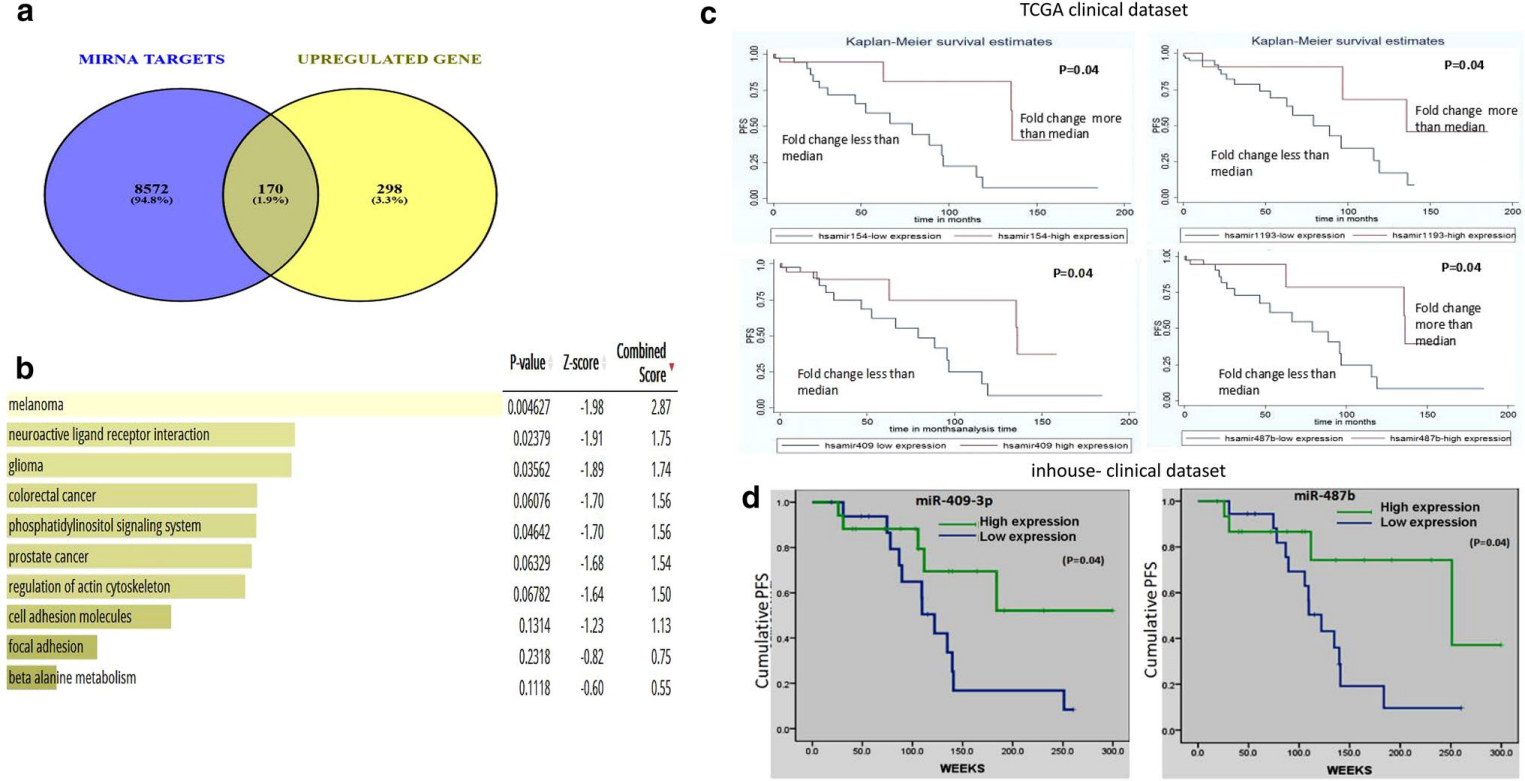
**a** Venn diagram showing genes common between the predictive directs and gene expression analysis. **b** Signalling pathway affected by C14MC dysregulation in ODGs. **c** Representative survival plot of the association between C14MC expression and patient outcome in ODGs using TCGA clinical dataset. Blue represents low expression, whereas red represents higher expression X- axis is time in months. **d** Kaplan Meier survival curve for patients for miR-409-3p and 487b using log rank test (inhouse clinical dataset). Patients that had lower expression level of miR-409-3p and miR-487b showed poor PFS than those which had higher expression of miR-409-3p and miR-487b (p = 0.04) for both

miRNAs exert their roles either through inhibiting or by degrading target mRNAs translation. This makes the status of miRNA target genes as a fundamental step to understand their biological functions. Hence, we evaluated the post-transcriptional regulatory network potentially regulated by C14MC miRNAs Insilco. For this we selected 43 downregulated miRNAs from the C14MC cluster and used the miRsystem webtool to search for predicted targets of each of these miRNAs. All the genes targeted by 43 miRNAs were included for Gene Set Enrichment Analysis (GSEA). Since the miRNAs from C14MC cluster showed downregulation in ODGs therefore, we compared the list of potential targets of these miRNAs with microarray mRNA expression data generated using human ST array chip and searched for those showing significant upregulation (> 1.5-fold) in ODG samples. The resulting list of 170 predicted targets was subsequently submitted to the EnrichR webtool to identify enriched biological processes and signaling pathways. The most significantly enriched biological processes were glioma pathway, notch signaling pathway and phosphatidylinositol signaling pathway (P value < 0.001) (Fig. 3a, b). These pathways have also been reported to be involved in ODGs in previous studies suggesting the role of C14MC downregulation in ODG pathogenesis by perturbing the tumorigenic and other biological processes [31, 32].

### Downregulation of C14MC miRNAs are associated with poor PFS in ODGs

Several studies have shown a tumor suppressor role of several downregulated miRNAs from C14MC by targeting key oncogenes in glioblastoma, pancreatic cancer, pulmonary cancer, glioneuronal tumors, metastatic lung cancer, has been well documented [33–36]. In contrast, miRNAs from the C14MC has been also reported to act as oncogenes as well [37] suggesting that these miRNAs may have different biological roles depending on the tissue of origin and genetic background. However, our survival analysis using TCGA clinical dataset revealed an association of downregulation of this cluster with poor progression free survival (PFS) in ODGs. Further survival analysis of the in-house clinical dataset using Kaplan–Meier curve also demonstrated similar finding. Higher fold under expression of miR-409-3p (Median fold change = − 3; p = 0.04) and miR-487b (Median fold change = − 2.8; p = 0.04) showed association with shorter PFS in total ODGs **(**Fig. 3c, d). However, we did not find significant association of C14MC with PFS separately in grade II and grade III ODGs. Similar to our observation, lower expression of miR-487b and miRNA-409-3p has also been reported to correlate with poor prognosis in several other tumors [16, 33–36]. This finding signifies that the optimal expression of the whole miRNA cluster could be linked to better prognosis, hence, highlighting on the tumor suppressor potential of the same.

## Conclusion

In conclusion, our work is the first to report the silencing of the second largest miRNA cluster in ODGs, importantly implicating its tumor-suppressive role in their pathogenesis. Dysregulation of the transcription factor (*MEF2*) along with epigenetic machinery involving DNA methylation and histone modifications could be the possible mechanism for C14MC downregulation, acting independently or in synergy, in ODGs. Overall, our findings have uncovered another layer of regulatory network of downregulation of C14MC and have increased our basic understanding of the genetic and epigenetics mechanisms responsible for oligodendroglioma genesis. However, future functional studies will shed additional light on the function of these miRNAs in ODG pathogenesis.

## Electronic supplementary material

Below is the link to the electronic supplementary material.

Supplementary material 1 (XLS 69 KB)
